# Metagenomics reveals the genetic diversity between sublineages of UCYN-A and their algal host plastids

**DOI:** 10.1093/ismeco/ycae150

**Published:** 2024-12-04

**Authors:** Ella Joy H Kantor, Brent M Robicheau, Jennifer Tolman, John M Archibald, Julie LaRoche

**Affiliations:** Department of Biology, Dalhousie University, Halifax, NS, Canada; Institute for Comparative Genomics, Dalhousie University, Halifax, NS, Canada; Department of Biology, Clark University, Worcester, MA, USA; Department of Biology, Dalhousie University, Halifax, NS, Canada; Institute for Comparative Genomics, Dalhousie University, Halifax, NS, Canada; Institute for Comparative Genomics, Dalhousie University, Halifax, NS, Canada; Department of Biochemistry & Molecular Biology, Dalhousie University, Halifax, NS, Canada; Department of Biology, Dalhousie University, Halifax, NS, Canada; Institute for Comparative Genomics, Dalhousie University, Halifax, NS, Canada

**Keywords:** UCYN-A, nitroplast, plastid, metagenomics, co-culturing microbes, ocean time series, pangenome

## Abstract

UCYN-A (or *Cand.* Atelocyanobacterium thalassa) has been recognized as a globally distributed, early stage, nitrogen-fixing organelle (the “nitroplast”) of cyanobacterial origin present in the haptophyte alga *Braarudosphaera bigelowii*. Although the nitroplast was recognized as UCYN-A2, not all sublineages of UCYN-A have been confirmed as nitroplasts, and full genomes are still lacking for several known sublineages. We investigated the differences between UCYN-A sublineages by sequencing and assembly of metagenomic sequences acquired from cultured biomass from NW Atlantic seawater, which yielded near-complete Metagenome Assembled Genomes (MAGs) corresponding to UCYN-A1, -A4, and the plastid of the UCYN-A4-associated *B. bigelowii.* Weekly time-series data paired with the recurrence of specific microbes in cultures used for metagenomics gave further insight into the microbial community associated with the algal/UCYN-A complex. The UCYN-A1 MAG was found to have 99% average nucleotide identity (ANI) to the Pacific-derived reference genome despite its Atlantic Ocean origin. Comparison of the UCYN-A4 MAG (the initial genome sequenced from this sublineage) to other genomes showed that UCYN-A4 is sufficiently genetically distinct from both UCYN-A1 and UCYN-A2 (ANI of ~83% and ~85%, respectively) to be considered its own sublineage, but more similar to UCYN-A2 than -A1, supporting its possible classification as a nitroplast. The *B. bigelowii* plastid sequence was compared with published plastid sequences (sharing 78% ANI with *Chrysochromulina parva*) adding to our understanding of genomic variation across Haptophyta organelles and emphasizing the need for further full genomic sequencing of *B. bigelowii* genotypes and their organelles.

## Introduction

Diazotrophic microbes play a key role in the marine nitrogen cycle by converting biologically unavailable atmospheric dinitrogen (N_2_) into ammonia, which can then be assimilated into organic nitrogen [1]. N_2_ fixation is accomplished by the multi-subunit nitrogenase enzyme [2]. The main gene used to assess the diversity and distribution of diazotrophs is *nifH,* which codes for the dinitrogenase reductase component of the nitrogenase enzyme [2–4]. Early sequencing of the *nifH* gene led to the discovery of a large clade of cyanobacterial diazotrophs that contribute significantly to global N_2_ fixation [5, 6] and whose members are either free-living or symbiotic with eukaryotic phytoplankton [7]. In particular, the symbiotic cyanobacterial diazotroph *Candidatus* Atelocyanobacterium thalassa (UCYN-A [8]) exhibits a cosmopolitan distribution and is associated with high N_2_ fixation rates [3, 9, 10]. The endosymbiotic lifestyle of UCYN-A was hypothesized early on due to its highly reduced genome and tight association with a haptophyte alga, with which it exchanges fixed nitrogen in return for plastid-derived fixed carbon [8, 11].

Up to eight UCYN-A sublineages—denoted UCYN-A1 to -A8—have been proposed through *nifH* metabarcoding and have also been considered ecotypes [12–14]. Evidence that UCYN-A is an early-stage nitrogen-fixing organelle, or “nitroplast,” was obtained using the *B. bigelowii*-associated UCYN-A2 ecotype, currently the only sublineage available in culture [15, 16]. Coale et al. [15] demonstrated synchronized division with the host and the presence of host nuclear encoded proteins in the UCYN-A2 proteome. These characteristics, paired with UCYN-A2 genome reduction [17] and the host-symbiont size relationship [16] provide strong evidence supporting the evolution of a novel organelle. We use the term UCYN-A to describe the *Candidatus* Atelocyanobacterium thalassa derived sublineages (i.e. UCYN-A1, -A2, etc.) to avoid confusion with prior publications that use this terminology/notation. Hereafter, UCYN-A2 represents the newly identified nitroplast of *B. bigelowii*.

Seven UCYN-A genomes are currently publicly available, three from the UCYN-A1 sublineage and four from UCYN-A2 (Table S1) [17–22]. The hosts of UCYN-A2 and -A1 have been identified by their differing cell sizes, unique *18S* rRNA genes, and specific CARD-FISH assays as *B. bigelowii* and a closely related prymnesiophyte, respectively [8, 14, 23, 24] (note the alternate life stage and synonym of *B. bigelowii* is called *Chrysochromulina parkeae* [24]). This study focuses on samples collected from the coastal Northwest Atlantic where Robicheau et al. [25] recently reported the occurrence of UCYN-A1, -A2, -A3, and -A4. Network analysis of the phytoplankton temporally associated with UCYN-A in this region found a strong co-presence of UCYN-A2 *nifH* gene signature with the *16S* rRNA plastid signature of the known host *B. bigelowii*, as well as a co-presence between UCYN-A1 and a differing *16S* rRNA plastid signature of a prymnesiophyte with sequence similarity to *Chrysochromulina* sp*.* [24, 25]. Although no complete plastid genome sequence of the host has been reported, three partial sequences of the UCYN-A2-associated *B. bigelowii* plastid have been published [15]. In addition, network analyses have shown co-occurrences of other phytoplankton to UCYN-A, such as *Synechococcus*, Dictyochophyceae, Syndiniales, and Gymnodiniales [25–27] and a recent study has shown evidence of selective grazing by *B. bigelowii* on co-occurring bacteria [28].

Here, we used culturing via selective nutrient addition to obtain UCYN-A within a mixed microbial community. Amplicon and metagenomic sequencing were used to characterize the microbial community of UCYN-A-containing cultures and to generate metagenome-assembled genomes (MAGs). We present a UCYN-A4 ecotype MAG and an associated *B. bigelowii* algal plastid sequence, as well as a pangenome analysis of currently available UCYN-A genomes. We further analyze the microbial community associated with UCYN-A both in cultures and in the natural waters of the coastal Northwest Atlantic using a weekly multi-year oceanographic time series.

## Materials and methods

### Water collection, culturing, and cell sorting

Seawater was collected via Niskin bottles from the offshore Scotian Shelf (at 20 m) in 2021 (Station HL7 during the 2021 Modular Ocean Research Infrastructure—Atlantic Condor Expedition; 42° 50′ 00” N, 61° 43′ 00” W) and weekly from the inshore Bedford Basin (at 5 m) during late summer/early fall of 2018 and 2020 (at 44° 41′ 37“ N, 63° 38’ 25” W; Halifax, N.S., Canada). The sampling period coincided with the observed presence of UCYN-A in this region [25]. Water samples were enriched with iron (FeCl_3_), phosphate (NaH_2_PO_4_), and vitamins (Biotin, Cobalamin, Thiamine-HCl; see Table S2 for concentrations) to select for diazotrophs under laboratory conditions (see [29, 30]). Incubations were carried out in polystyrene tissue culturing flasks (Greiner Bio-One, Austria) at 15°C and 12 h light/dark cycles for at least two weeks to allow for the stabilization of the community prior to further experimentation (Table S2). Following this initial incubation, cultures were screened using quantitative-PCR (qPCR) *nifH* assays for UCYN-A1 or -A2/-A3/-A4 using DNA extracted from filtered cells [25, 31]–noting that the UCYN-A2 assay used is cross-reactive to -A3 and -A4 [25, 32]. Cultures with high UCYN-A *nifH* counts were retained for further analysis.

One sample from the Scotian Shelf (Shelf 1) and one sample from the Bedford Basin (Basin 1) had a high abundance of UCYN-A1 directly following initial nutrient amendment and multi-week incubation; cells from these samples were directly used for DNA extractions and sequencing without cell sorting (Table S2). Other samples containing UCYN-A2/A3/A4 were subjected to fluorescence-activated cell sorting (FACS) using a BD Influx Cell Sorter to further concentrate cells containing UCYN-A for additional culturing/collection. Sorted cytogram populations were screened using the qPCR assays used above [25, 31]. For the other Bedford Basin sample (Basin 2) we sorted 2000 cells from a cytogram gate attributed to a UCYN-A/haptophyte complex into 0.2 μm filter-sterilized culture medium (Fig. S1A–C). Note that larger particles generally greater than 3 μm with a positive chlorophyll signature were sorted and therefore the sorting strategy presumably targeted the algal/UCYN-A complex. Sorted cells were immediately transferred and grown for another 8.5 weeks in ~10 ml of 0.2 μm-filtered Bedford Basin seawater taken at the time of sample collection. This seawater was further enriched with Fe, PO_4_, and vitamins after 0 weeks, 2 weeks, and 4 weeks of secondary incubation (concentrations as in Table S2). For the Scotian Shelf sorted sample (Shelf 2) that contained mainly UCYN-A2/A3/A4, we did not have to re-culture sorted cells to achieve a high biomass for downstream molecular work. Instead, we used ~28 000 sorted cells attributed to a UCYN-A containing cytogram population (qPCR-screened) for direct DNA extraction and sequencing (Fig. S1D–F). Cultures not subjected to cell sorting were harvested for molecular work by filtration onto 0.2 μm polycarbonate Isopore filters to collect biomass from 30-50 ml of culture; alternatively, we used sorted cells directly.

In addition to seawater collected especially for culturing, weekly Bedford Basin time-series seawater was used for microbial community composition analyses via 16S rRNA metabarcoding. We use the term “cultures” throughout to specify the difference between datasets that were derived from longer-term incubations and/or cell sorting (note these cultures are not axenic), and those of the natural *in-situ* community. Methods used in collecting Bedford Basin time-series water samples for *in-situ* work were as previously described [33].

### DNA extractions and sequencing

DNA was extracted using a DNeasy Plant Mini kit according to the manufacturer’s instructions (Qiagen, Germany) along with a modified lysis procedure [34]. Where specified, DNA was sent for metagenomic sequencing on an Illumina NextSeq instrument (Table S3) and for metabarcoding of the 18S rRNA V4 variable region (primers E572F & E1009R [35]) and the 16S rRNA V6-V8 variable regions (primers B969F & BA1406R [35]) via Illumina MiSeq at the Integrated Microbiome Resource at Dalhousie University (Halifax, N.S., Canada) [36]. Downstream analysis on amplicon sequencing data was done using the same methods and pipelines as in Robicheau et al. [33].

### Data analyses

Metagenomic sequences were processed into bins and MAGs using the metagenomic workflow in Anvi’o v7.1 as eight sequencing runs in one set (Table S3) [37, 38]. Commands used to process raw reads into a contigs database with open reading frames (ORFs) identified are given in Supplementary Methods S1. The final contigs database that includes ORFs was populated with annotations, first with the identification of single-copy core genes (SCGs) using Hidden Markov Models by HMMR [39]. SCGs were taxonomically classified using The Genome Taxonomy Database [40]. The gene calls were classified taxonomically using Kaiju [41] and functionally annotated using NCBI’s COG, Pfam protein family database, and the KOfam database of KEGG orthologs [42–44]. Sorting and indexing of the BAM files from the mapping was done using SAMtools [45]. The mapping results were profiled using “anvi-profile”, which characterizes properties of every contig in a sample such as coverage and single nucleotide variants into a profile database; the profile databases for each sample were merged into one. Automatic binning was done initially using METABAT2 [46] then manually adjusted using the Anvi’o interactive interface to separate bins with high redundancy following the online tutorial [47, 48]. 104 bins were assembled from culture metagenomic data, of which 35 were classified as MAGs if they met the threshold of over 70% completion or over 2 megabase pairs (Mbp) in size, and below 10% redundancy.

The pangenome was analyzed using the Anvi’o pangenomics workflow [49, 50]. UCYN-A genomes used include the two UCYN-A MAGs generated herein via Anvi’o and seven other published genomes which we refer to by the names given in Table S1. Prior genomes include the complete reference genome for UCYN-A1 ALOHA of Tripp et al. [18] and the reference UCYN-A2 CPSB-1 genome (the nitroplast) of Suzuki et al. [21]. The UCYN-A3 genome is partial, so only *16S* rRNA and *nifH* genes were used for this analysis [51]. Genomes were first uploaded into Anvi’o v7.1 and a contigs database was created for each genome and populated using the annotation steps given above and in Supplementary Methods S1, excluding Kaiju taxonomy. The commands used to generate the pangenome database, the calculation of average nucleotide identity (ANI) and pangenome visualizations are given in Supplementary Methods S2.

A MAG for the plastid genome of *B. bigelowii* was assembled using only the sequences from the Shelf 2 sample. This sample was run through the same Anvi’o metagenomics workflow described earlier except all binning was done manually. The published plastid genome of *Chrysochromulina parva* (accession NC_036937.1 [52]) was used as a reference and the bins containing a GC content similar to this plastid genome (~30% GC) were pulled for further examination. Contigs with high BLAST similarity to the *C. parva* reference were used as scaffolds on which to carry out further assembly and mapping of the raw reads using Geneious Prime 2023.2.1 (https://www.geneious.com). Plastid genome annotations shown herein, as well as synteny and alignment dotplots were determined using Supplementary Methods S3.

16S rRNA amplicon data were processed according to Supplementary Methods S4. Geneious Prime 2023.2.1 (https://www.geneious.com) was used to locally align (via BLAST [53]) the *nifH* genes of the MAGs against UCYN-A *nifH* ASVs in Robicheau et al. [25] and oligotypes in Turk-Kubo et al. [12], with the goal of assigning UCYN-A genomes to specific sublineages. The N_2_ fixation gene region was identified using gene annotations and the Nif operon described by Zehr et al. [54]. Clustal Omega v1.2.3 [55] was used to align the nitrogenase coding regions across genomes and to align 16S and 18S rRNA sequences from the metagenomic and amplicon sequencing data.

Additional genome annotation was done using the RAST annotation service [56–58]. Proksee was used for visualization of genomes and BLAST results [59]. MAUVE v1.1.3 was used to align MAGs to reference genomes to compare genes [60]. CheckM2 v1.0.1 [61] was used to calculate genome completeness and contamination in addition to the tool built into Anvi’o; CheckM2 results are presented due to its higher accuracy in predicting genome quality for reduced genomes. Completeness and redundancy predictions for all nine genomes were done using the “checkm2 predict” command and used the specific neural network model [61].

## Results

### Community composition of cultured seawater

**Figure 1:**
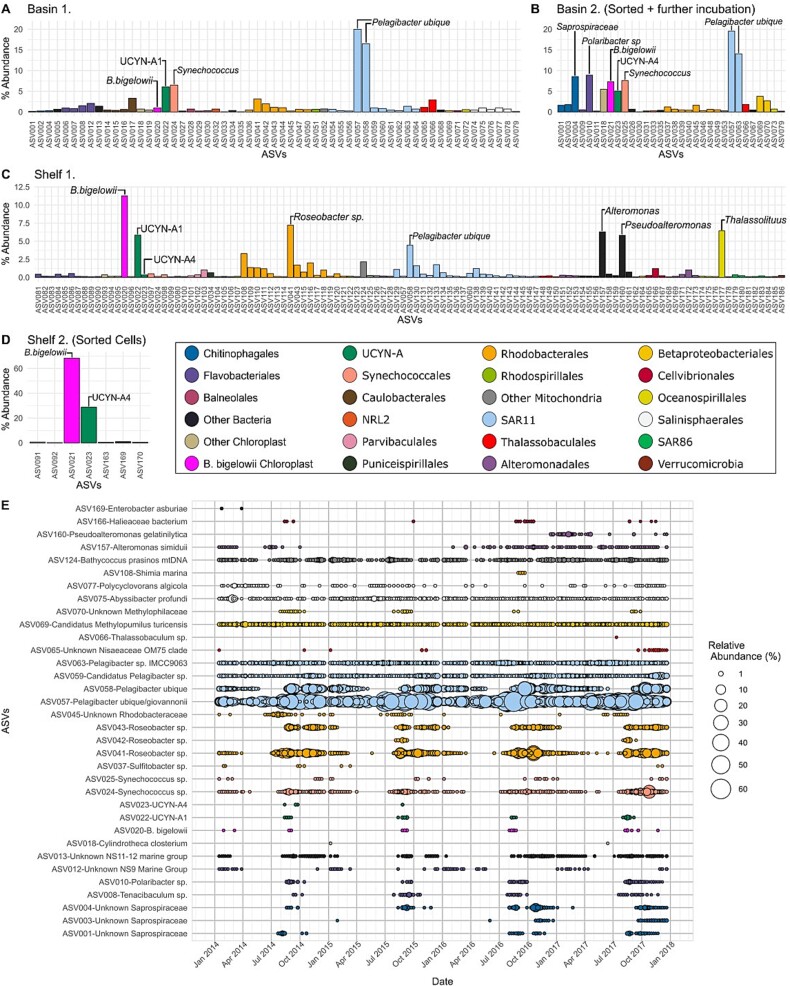
Community characterization of enrichment cultures based on relative abundance of ASVs. The results of the 16S rRNA amplicon sequencing, ASVs with ≥0.1% relative abundance are plotted for each culture used in the analyses (A–D). Panel E uses 16S rRNA data from the Bedford Basin weekly time series to show percent abundance in the Bedford Basin of ASVs with ≥1% abundance in the cultures for the 5 m depth from the years 2014–2017. (additional information on cultures is provided in Table S2 and on ASVs in Table S4).

Though not axenic, UCYN-A cultures had a lower species richness than natural water collected from the Bedford Basin and had a relatively higher proportion of UCYN-A either from shifting the community towards diazotrophs and/or due to the sorting procedures that targeted UCYN-A containing cells (Fig. 1). The total number of 16S ASVs found in the cultures ranged from seven to 115, while the number of ASVs found on average in a natural water sample is 374. UCYN-A *nifH* qPCR counted ~1.9 × 10^8^ copies per liter of UCYN-A1 in the Shelf 1 culture and ~1.9 × 10^9^ copies per liter of UCYN-A2 in the Shelf 2 culture. In the Bedford Basin time series from 2014–2017 for all depths combined the UCYN-A1 reached a maximum of ~3.5 × 10^6^ copies per liter and UCYN-A2 reached a maximum of ~4.5 × 10^6^ copies per liter [25], up to three orders of magnitude lower than what was found in the cultures. 16S rRNA V6-V8 metabarcoding of cultures yielded two unique UCYN-A ASVs (ASV022 + ASV023) across the four cultures, as well as two unique ASVs for *Braarudosphaera bigelowii* plastids (ASV020 + ASV021) (Fig. 1A–D). The cell-sorted Shelf 2 sample had the lowest total ASV richness; this sample consisted mainly of sequences from the *B. bigelowii* plastid and UCYN-A4 (Fig. 1D), which together accounted for more than 90% of the sequence reads. In other samples some taxa occurred at proportions comparable to the *B. bigelowii*/UCYN-A ASVs, indicating they were co-occurring during *ex situ* culturing (Fig. 1A–C). Such ASVs belonged to *Pelagibacter ubique, Polaribacter* sp.*, Synechococcus* sp.*,* and an unknown Saprospiraceae in Basin cultures (Fig. 1A, B). For the non-sorted Shelf 1 culture, *Roseobacter* sp., *Thalassolituus* sp., *Alteromonas* sp.*, Pseudoalteromonas* sp.*,* and *Pelagibacter ubique* were co-occurring (Fig. 1C). Identical ASVs corresponding to those detected for the taxa mentioned above were also detected in the 4-year weekly Bedford Basin 16S rRNA time-series dataset (Fig. 1E). Some of the taxa occur in the Bedford Basin throughout the year irrespective of UCYN-A (e.g., ASV075 *Abyssibacter profundi*), whereas for others (e.g., *Roseobacter* sp.) their seasonal presence roughly coincides with that of *B. bigelowii*/UCYN-A (Fig. 1E). In general, the most abundant microbes in the cultures also had high overall relative abundances in the Bedford Basin (Fig. 1E). Although some of the broader taxonomy for the total set of ASVs that co-occurred overlaps across samples (for those ASVs with >0.1% relative abundance), there was very little overlap at the ASV level between co-occurring taxa from the Shelf samples and the Basin samples (Fig. S2, Table S4).

Since they had fewer ASVs, the Basin 2 and Shelf 2 samples were sequenced for 18S rRNA to further refine any putative haptophyte signatures associated with UCYN-A (see Table S5A). One ASV was present in both cultures and was taxonomically classified as *B. bigelowii*; it was the most abundant 18S rRNA ASV in the Shelf 2 sample and the second most abundant in Basin 2 (Table S5A). This *B. bigelowii* 18S rRNA ASV is identical to an aligned portion of 18S rRNA of multiple sequences, all classified as genotype I, and derived from the Northwest Pacific Ocean [62, 63] (Shukutsu22, Shukutsu27, Shukutsu19, and TP05–6-b; Table S5B, C). This highlights, for the gene region in question, that nucleotide similarity can be identical across Pacific and Atlantic Ocean basins.

### MAGs of UCYN-A from the coastal Northwest Atlantic and *nifH* gene analysis

**Figure 2:**
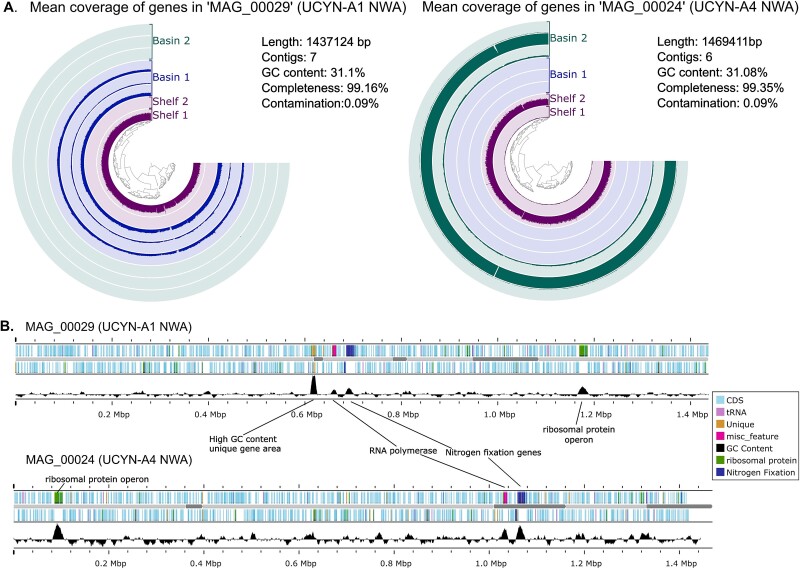
The two UCYN-A MAGs assembled from the metagenomic sequencing of cultured seawater. (A) The average coverage of genes within each bin by sample shows UCYN-A1 NWA had a higher proportion of reads mapped from the Basin 1 and Shelf 1 samples. UCYN-A4 NWA showed a higher proportion of reads mapped from Basin 2 and Shelf 2 samples. (B) Annotations of UCYN-A1 NWA and UCYN-A4 NWA show areas of higher GC content aligning with the nitrogenase genes, two large RNA polymerase subunits, and a ribosomal protein operon.

Two MAGs, MAG_00024 and MAG_00029, belonged to UCYN-A genomes based on SCG taxonomy identification. MAG_00029 is 1.44 Mbp in seven contigs and is mostly composed of reads from Basin 1 and Shelf 1 samples (Fig. 2). MAG_00024 is 1.47 Mbp in six contigs and is dominated by reads from the Basin 2 and Shelf 2 samples (Fig. 2). CheckM2 results estimated 99.16% completeness and 0.09% contamination for MAG_00029 and 99.35% completeness and 0.08% contamination for MAG_00024 (Table S6).

The *nifH* genes in both MAGs aligned against other UCYN-A *nifH* ASVs and oligotypes indicate MAG_00029 has 100% pairwise identity with the UCYN-A1 ASV “A1-deb” [25] and the oligotype “Oligo_1” [12]. The *nifH* gene from MAG_00024 has 100% pairwise identity with UCYN-A4 ASV “A4–511” [25] and oligotype “Oligo_4” [12]. Hence, *nifH* gene analysis shows our culturing and metagenomics was successful in obtaining UCYN-A1 and UCYN-A4 genomes from the coastal Northwest Atlantic (the first reported genome of the UCYN-A4 sublineage) and further results on genome comparison support these findings. MAG_00029 and MAG_00024 will subsequently be referred to as genomes UCYN-A1 NWA and UCYN-A4 NWA, respectively.

### The UCYN-A pangenome

**Figure 3:**
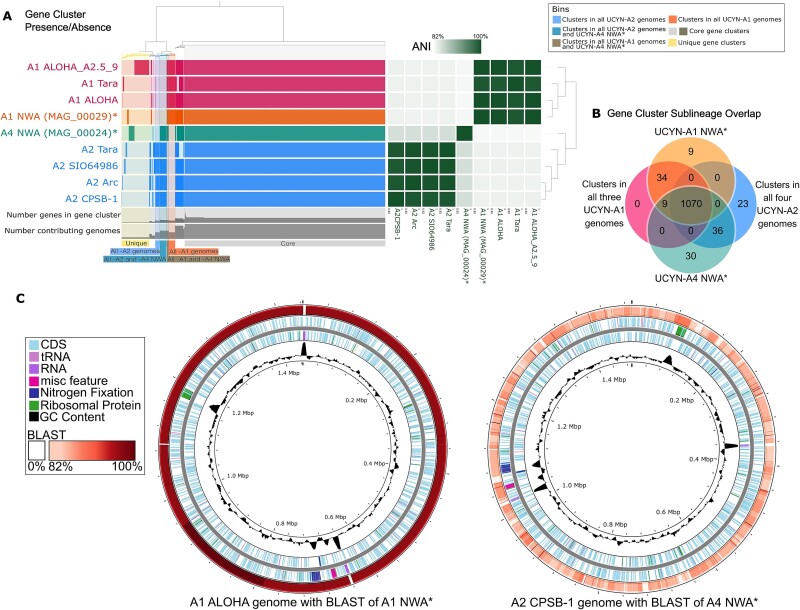
Pangenome analysis of UCYN-A. (A) Anvi’o schematic showing presence/absence of gene clusters in each genome (left) and ANI between each genome. Bins (with the exception of unique and core gene clusters) are color coded by their corresponding sections in (B). There are 337 peripheral gene clusters that were (not core) and 147 of them are unique to one genome. (B) The Venn-diagram of gene clusters in the MAGs that overlap with the clusters that are in all three published UCYN-A1 genomes and all four published UCYN-A2 genomes. The center of the Venn-diagram represents the 1070 core gene clusters which are present in all nine genomes (color was changed in (A) for visibility). (C) Full UCYN-A1 reference genome (A1 ALOHA) annotated with BLAST results against UCYN-A1 NWA* and full UCYN-A2 reference genome (A2 CPSB-1) annotated with BLAST results against UCYN-A4 NWA* (* = from this study).

Pangenome analysis revealed a high level of intra-sublineage conservation. The ANI was >99% between the three published UCYN-A1 genomes and between the four published UCYN-A2 genomes (Fig. 3A; Table S7). In contrast, the ANI between UCYN-A1 versus -A2 was between 83.2–83.4%. The UCYN-A4 NWA genome had 82.6–82.7% ANI with the three UCYN-A1 genomes and 85.2–85.3% ANI with the four UCYN-A2 genomes (Table S7), hence, the UCYN-A4 NWA genome is as different from both UCYN-A1 and -A2 as these two are from each other with higher similarity to UCYN-A2 than -A1.

Analysis of gene cluster presence/absence resulted in 1407 total gene clusters: 1070 “core” gene clusters (found in all nine genomes) and 337 peripheral gene clusters (Fig. 3A). 147 of the peripheral gene clusters were unique to one genome (Fig. 3A) and all but one contained a single gene (due to naming conventions in Anvi’o, note that in the analysis “clusters” can contain a single gene) [49]. The genome with the most unique genes was UCYN-A1 ALOHA_A2.5_9 with 79 unique genes which were likely partial genes that were created because the genome is in 47 contigs; the rest of the published genomes had between one and eight unique genes (Fig. 3A). UCYN-A4 NWA has 30 unique gene clusters and genes, and the UCYN-A1 NWA has nine unique gene clusters and 10 unique genes (Fig. 3A, B). Looking at genomes of the same sublineage, there were 34 gene clusters exclusive to the four UCYN-A1 genomes (including the UCYN-A1 NWA) and 23 gene clusters exclusive to the four UCYN-A2 genomes (Fig. 3B). Therefore, the 30 gene clusters unique to UCYN-A4 NWA fall within observed range for other sublineages despite having only one representative genome, indicating a similar amount of genetic dissimilarity between the UCYN-A4 sublineage and the other characterized sublineages of UCYN-A1 and -A2. UCYN-A4 NWA does, however, share more gene clusters with UCYN-A2 genomes than it does with UCYN-A1 genomes (36 gene clusters versus nine gene clusters; Fig. 3B), which supports the same pattern of similarity seen with the ANI results.

Of the 10 genes unique to UCYN-A1 NWA, five exhibit high sequence similarity to partial genes, hypothetical protein genes, or unannotated regions in UCYN-A1 ALOHA (Table S8). These genes were likely labeled as “unique” because they were partial due to the nature of MAG creation, and not recognized by the algorithm. Two of these unique genes had no annotation in Anvi’o and have no obvious counterparts using BLAST. The other three genes were annotated as a helicase conserved C-terminal domain coding region and two swr1 complex snf2 family DNA-dependent ATPases. These three genes show BLAST similarity with partial proteins from the genomes of the haptophytes *Gephyrocapsa huxleyi* (formerly *Emiliania huxleyi*) and *Chrysochromulina tobinii* and were located in a high GC content (48%) area at the ends of two contigs with seven unique genes and are, therefore, likely to be artefacts of the assembly process and not true genes unique to the genome (Fig. 2B)*.* BLAST results of the UCYN-A1 NWA against the UCYN-A1 ALOHA reference genome showed high percent identity of over 98% in most regions except for missing rRNA genes (Fig. 3C).

Of the 30 gene clusters unique to the UCYN-A4 NWA, only four were annotated by Anvi’o. Of the rest, three have BLAST matches to known proteins in public databases, 11 were annotated as hypothetical proteins, and 12 had no annotations beyond their original identification as ORFs (Table S9). Three of the genes with annotations are adjacent at the ends of two contigs: a *MalK* maltose/maltodextrin ATP-binding protein, a 23S rRNA-intervening sequence protein with BLASTx match to a four-helix bundle protein of unknown function from *Gracilimonas* sp*.*, and a gene with a BLASTx match to a *HlyD* family efflux transporter from *Balneola* sp. The *MalK* has 93% identity in BLASTn with a portion of the same gene in the UCYN-A2 CPSB-1 genome, while the other two had no BLASTn results and do not align with UCYN-A2 CPSB-1. Other annotated genes encode an RNA polymerase sigma factor with BLASTx match to *Crocosphaera* sp*.*, a glycosyltransferase *BscA*, a partial ferredoxin gene which partially aligns to the same gene in UCYN-A2 CPSB-1, and DUF3086 domain-containing protein of unknown function with a weak BLASTx match to the same protein in UCYN-A2 CPSB-1 (45% amino acid identity). Five hypothetical protein genes and one unannotated gene were found to align with regions in UCYN-A2 CPSB-1 with no annotations. A BLASTn of UCYN-A4 NWA against UCYN-A2 CPSB-1 showed an average identity in the low 80% range and was also missing rRNA genes (Fig. 3C).

### Nitrogenase and 16S rRNA coding region comparison

**Figure 4:**
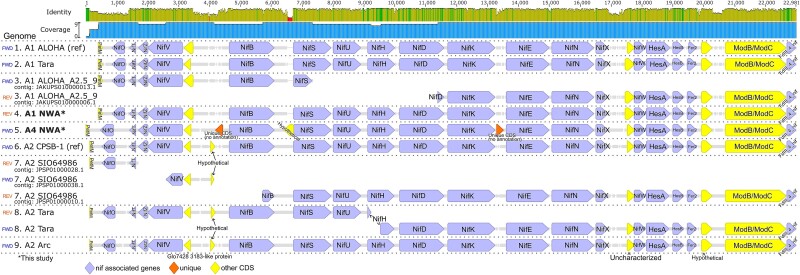
*Nif* gene region alignment of all genomes includes the region between *PetM* and *FdIII_4_nif* genes chosen because they were present in all genomes. Genomes with regions spanning multiple contigs were aligned using each contig separately (except for the A1 Tara region which spanned two contigs that overlapped and were able to be merged).

Although the *nif* gene regions across sequenced genomes are largely syntenic (Fig. 4), there are obvious differences that are likely due to a lack of complete genomes in some cases (e.g., a missing *nifH* gene in the UCYN-A1 ALOHA_A2.5_9 genome; see Supplementary Results S1 for specific details). Other differences unique to UCYN-A4 NWA are three hypothetical proteins that are not present in any other genomes (Fig. 4).

Two different 16S rRNA ASVs for UCYN-A were obtained by amplicon sequencing of our cultures: ASV022 (Basin 1 and Shelf 1) and ASV023 (Basin 2 and Shelf 2; Fig. 1A-D). The 16S rRNA genes from known genomes, as well as the published UCYN-A3 16S rRNA gene (MH807559) [51] and the UCYN-A2 16S rRNA gene from isolate TMRscBb7 (AB847982) [14, 24] were aligned allowing comparison of the 16S rRNA gene across sublineages (Table S10). In UCYN-A1 ALOHA, -A2 SIO64986, and -A2 CPSB-1 there were two copies of the rRNA genes, one forward and one reverse. The 16S genes from A2 CPSB-1 were not 100% identical to each other. The forward 16S gene in A2 CPSB-1 had the lowest identity to all other 16S genes in the analysis while the reverse 16S gene had 99% identity with other UCYN-A2 16S rRNA genes. In the UCYN-A1 genes, ASV022 from Basin 1 and Shelf 1 samples matched perfectly to UCYN-A1 ALOHA and UCYN-A1 ALOHA_A2.5_9 (Table S10). ASV023 from Basin 2 and Shelf 2 samples was not 100% identical to any other 16S rRNA ASVs from published UCYN-A genomes; it was, however, 99.2% identical to the 16S rRNA from the UCYN-A2 sequences and the UCYN-A3 16S gene (Table S10).

### The plastid genome of *B. bigelowii* associated with UCYN-A4

**Figure 5:**
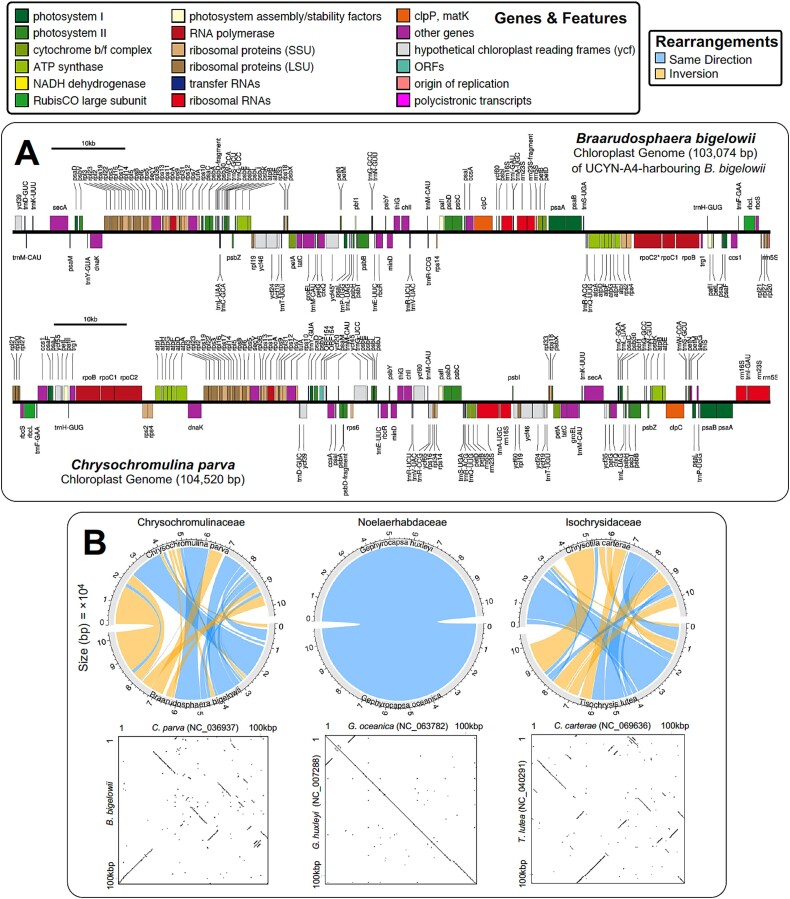
The *Braarudosphaera bigelowii* plastid NWA genome annotated*,* shown above a previously published *Chrysochromulina parva* chloroplast genome (A). The *B. bigelowii* genome has many of the same genes as the *C. parva* but is most noticeably missing the second rRNA operon which is difficult to assemble from metagenomic sequencing due to it being duplicated in the genome. (B) Visualization of genome rearrangements and synteny between various haptophyte chloroplast genomes including MAUVE alignments (upper) that identifies homologous regions or “Locally Collinear Blocks”, as well as alignment dotplots at a sliding window-size of 100 bp (lower). The ANI between *B. bigelowii* and *C. parva* is 78%.

The *B. bigelowii* plastid genome (named here: *B. bigelowii* plastid NWA) was assembled into a single contig of 103 074 bp. B. bigelowii plastid NWA is only 1.5 kbp smaller than the published plastid genome of *C. parva* (accession NC_036937 [52]), a related species within the same genus (Fig. 5A) with the main difference being a missing inverted repeat of the rRNA operon in *B. bigelowii* plastid NWA. Much of the published *C. parva* plastid genome is accounted for in homologous regions of the *B. bigelowii* plastid NWA genome, with an overall 78% ANI between the *B. bigelowii* plastid NWA and *C. parva* plastid genomes, but there are many large-scale rearrangements, and some small unique regions exist in both assemblies (Fig. 5B). This is not unexpected; inspection of currently available complete haptophyte plastid genomes in NCBI reveals substantial variation in the level of synteny (gene order conservation) between species, from high levels (e.g., *G. huxleyi* versus *Gephyrocapsa oceanica* within the Noelaerhabdaceae) to a comparably lower level of synteny within the *Chrysochromulina* (e.g., *Chrysotila carterae* versus *Tisochrysis lutea* within the Isochrysidaceae) (Fig. 5B); recall that *C. parkae* and *B. bigelowii* are considered synonyms [24]. Alignment of the 16S rRNA derived from the *B. bigelowii* plastid NWA genome also suggests that intraspecific variation exists in this gene region within *B. bigelowii* (Supplementary Results S2; Table S11). BLAST alignment of *B. bigelowii* plastid NWA with the recently published plastid contigs from the *B. bigelowii* hosting UCYN-A2 (OR912955.1, OR912954.1, OR912953.1) [15] while accounting for ~73% of the whole plastid genome, showed 90–91% identity (90–93% ANI) over partial regions of alignment (~74 000 bp) (Fig. S4).

## Discussion

### The UCYN-A4 genome is distinct from both the UCYN-A1 and UCYN-A2 genome, and its putative host is *B. bigelowii* genotype I

The sequencing and assembly of MAGs is a powerful tool for exploring genomic diversity but can be problematic when closely related strains co-exist in samples [64]. Pangenome results suggest that this is not the case for the UCYN-A MAGs presented herein and give evidence that they are genomes of separate sublineages, UCYN-A1 and UCYN-A4. UCYN-A1 NWA is effectively indistinguishable from UCYN-A1 ALOHA even though they are from different ocean basins (Pacific versus Atlantic); these results support findings of previous sublineage comparisons [22]. Unique genes in UCYN-A1 NWA occurred at the ends of contigs and therefore may be erroneous due to lower coverage or repeats such as where the rRNA operons are missing. RNA genes are difficult to assemble and bin into MAGs since they are highly conserved, have different GC content, and often have multiple copies in a genome [65].

Although UCYN-A2 is known to be prevalent in the coastal NW Atlantic and can co-occur with UCYN-A4 [25], we did not detect it in the amplicon sequencing of our cultures, nor did we detect the UCYN-A2 host *B. bigelowii* ASV. The ANI comparison of UCYN-A4 to -A1 and -A2 of 82–85% placed them within the range where 95% ANI in bacteria is considered intrageneric yet interspecific [66]. However, if UCYN-A is to be treated as an organelle instead of a cyanobacterium then the intraspecies diversity of organelles should be considered. The ANI between the *B. bigelowii* plastid genomes is 90%—higher than for the UCYN-As—but plastid genetic diversity between what are described as members of the same species can range from 99% ANI between two strains of the haptophyte *Phaeocystis globosa* (MT471334.1 and MT471331.1 [67, 68]) to 79% sequence identity between two strains of *Cryptomonas curvata* [69]*.* UCYN-A diversity is thus quite high, and multiple metrics comparing UCYN-A4 NWA to other UCYN-A genomes (ANI, the number of unique genes, and the *nif* gene coding regions; Figs. 3-4) together support UCYN-A4 NWA as a distinct sublineage especially because its genome is as different from both UCYN-A1 and -A2 as they are from each other.

The UCYN-A4 NWA genome is, however, slightly more similar to the UCYN-A2 ecotype than the UCYN-A1 ecotype. Previous work based only on the UCYN-A *nifH* phylogeny in the Bedford Basin did not capture the closer phylogenetic relationship between these sublineages [25]. In this study, however, full genome comparison, the *nif* comparisons, and pangenomics support the results of the 16S rRNA metabarcoding of the cultures which show that UCYN-A4 is more genetically like UCYN-A2 than -A1 (Fig. S2). One reason for why this similarity exists may be related to the co-occurrence of UCYN-A2 and -A4 that has been observed in coastal waters [12, 25]. Another reason for this similarity may be in the evolutionary patterns of UCYN-A and its host.

With fewer host sequences, it is harder to see a pattern of evolution, but if the symbiotic partnership between the two organisms is obligate, one would expect the evolution of the host to mirror that of UCYN-A. The presence of a corresponding *B. bigelowii* 16S rRNA ASV, 18S rRNA ASV, and near-complete plastid genome in sorted enrichment cultures alongside UCYN-A4 NWA supports the hypothesis that a *B. bigelowii* algae of Genotype I hosts the UCYN-A4, and these genetic signatures are likely evolutionary pairs, while the known host of UCYN-A2 is B. bigelowii genotype III [21, 24]. Meanwhile, our samples with UCYN-A1 contained a different *B. bigelowii* plastid 16S rRNA sequence (ASV020), pointing also to a *B. bigelowii* genotype as the possible host signature for the UCYN-A1 NWA identified herein different from the UCYN-A2 and -A4 hosts. This 16S rRNA ASV is different from the *Chrysochromulina* sp*.* plastid ASV found commonly co-occurring in the Bedford Basin with UCYN-A1 [25]. Whether these plastid 16S ASVs correspond to the 18S sequences of the known host of UCYN-A1 [8] has yet to be determined because of the lack of studies connecting the 18S with the plastid 16S sequences of *B. bigelowii.* A phylogenetic tree published comparing the 18S sequences of the hosts of both UCYN-A1 (FJ537341) and UCYN-A2 (AB250784) as well as the putative host of UCYN-A4 (AB250785) [24] showed a closer grouping of the UCYN-A2 and -A4 hosts than the UCYN-A1 host which is placed separately at the base of that clade. Thus, this closer genetic similarity between UCYN-A2 and UCYN-A4 may mirror the phylogenetic relationships found between their hosts. Studies have shown, however, that *B. bigelowii* has been observed without UCYN-A [21, 70], so it is possible that the relationship is not obligate and therefore the mirroring of host-endosymbiont evolution would not occur. Either way, this kind of comparison is only possible using full genomes of UCYN-A and host identification and genomics which can be improved upon with the isolation of more sublineages of UCYN-A and genotypes of *B. bigelowii.*

### Co-cultured microbial groups

Microbes present at high relative abundances in the cultures represent those occurring when *B. bigelowii* and UCYN-A are present in their natural habitat as well (Fig. 1E). Though the ASVs were not identical between cultured samples, the similarity of co-cultured organisms at higher taxonomic levels (e.g., genera) gives some initial insight into which microbial groups might occur together with *B. bigelowii*/UCYN-A. For example, some haptophytes are known to be mixotrophic [71, 72], and prior studies support the idea that *B. bigelowii* is capable of phagocytosis both as the mechanism for it acquiring UCYN-A originally as an endosymbiont and for acquiring nutrients [21, 28, 73]. Though grazing rate measurements were not done with our cultures, recent experimental results showed that *B. bigelowii* grazes on co-cultured bacteria including orders also found in our cultures such as Chitinophagales, Rhodospirillales, and Flavobacteriales [28]. These results suggest that some of the microbes that co-cultured with UCYN-A may be a food source for mixotrophic *B. bigelowii* [16, 28], which may have in-turn helped this haptophyte remain in our cultures for multiple weeks*.*

An alternative explanation is that these microbes have co-cultured alongside *B. bigelowii* simply because their growth was stimulated by the same temperature and lighting conditions and/or they may have been stimulated via the use of carbon and nitrogen that would have theoretically been fixed by *B. bigelowii*/UCYN-A present in the cultures (note we did not add nitrogen or carbon nutrients to any of our incubated seawater [74]). Given that some of the same bacteria were retained after targeting larger eukaryotic cells within a cytogram population containing UCYN-A during FACS, these microbes may have also been physically attached to larger cells, and therefore co-sorted as single events/particles (e.g., *Pelagibacter ubique*; Fig. 1B, S2).

Future studies aimed at better resolving specific interspecies dynamics and metabolic interactions between *B. bigelowii* and other marine microbes (e.g. through mixotrophy or autotrophy) may shed light on the observed associations. This could facilitate future efforts to create an axenic culture and to cultivate other *B. bigelowii*/UCYN-A ecotypes; given the vast amount of microbial diversity present in the ocean [75], having a narrower list of microbes known to co-culture *ex situ* and/or co-occur in the natural environment with *B. bigelowii–*like those mentioned herein and also identified from prior network analyses [25–27, 76]–can help inform the selection of microbial strains for this type of future research.

### UCYN-A4 as a possible nitroplast

UCYN-A2 was classified as a nitroplast because it has key characteristics: a reduced genome [17], a volumetric relationship with its host similar to that observed in organelles [16], cell architecture integration and synchronous division with other organelles, and imported proteins from the host [15]. UCYN-A4 NWA has similar genome reduction that is characteristic of UCYN-A with a length ~1.4 Mbp. Annotated genes unique to UCYN-A4 NWA were mostly hypothetical genes that align only partially to the UCYN-A2 reference genome or were located on the ends of contigs which are error prone regions due to the assembly process (Table S9). Protein import is a defining feature of an organelle relative to an endosymbiont [77]. Such proteins imported into the nitroplast from the host complemented key steps in biological pathways that are missing genes in the UCYN-A2 genome because of genome reduction [15]. These missing genes in UCYN-A2, the proposed nitroplast [15], are also missing in the previously published UCYN-A1 reference genome [54] and from UCYN-A4 NWA. This supports the idea that host-encoded proteins may be filling in these gaps in the other sublineages as well and therefore the -A1 and -A4 sublineages are likely nitroplasts currently or are on their way to becoming nitroplasts.

Relative to the endosymbiotic origins of mitochondria and plastids from alphaproteobacteria and cyanobacteria respectively [78], the UCYN-A2 nitroplast of *B. bigelowii* appears to be at a much earlier stage of organellogenesis, like that seen in the chromatophore of the testate amoeba *Paulinella* [15, 79]. Phylogenomic studies suggest that the common UCYN-A ancestor was already associated with an ancestor of *B. bigelowii*, and had undergone genome reduction, before the UCYN-A sublineages diverged ~90 million years ago [17, 23]. It is unclear at what stage of organellogenesis UCYN-A was at the time of divergence and unclear now as to whether the sublineages of UCYN-A are all at the same stage. With further study, the comparison of the plastid and nuclear genomes between hosts could be conducted in parallel with the comparison of UCYN-A/nitroplast genomes and opens the door for studying the details of their evolution and divergence. Using new culturing techniques plus long-read sequencing, the full nuclear and organellar genomes of different genotypes could be compared to determine the details of each UCYN-A-containing *B. bigelowii* lineage.

## Supplementary Material

Supplementary_title_text_tables_and_figures_ycae150

tableS4_ycae150

tableS5_ycae150

tableS8_S9_ycae150

## Data Availability

Amplicon and metagenomic sequencing files are available on GenBank under the BioProject PRJNA1132261 with BioSamples SAMN42337743, SAMN42337744, SAMN42337745, SAMN42337746 for Basin 1, Basin 2, Shelf 1, and Shelf 2, respectively. UCYN-A1 NWA, UCYN-A4 NWA, and *B. bigelowii* plastid NWA are in that same BioProject PRJNA1132261.
