# Supplementary material for: Metagenomics reveals the genetic diversity between sublineages of UCYN-A and their algal host plastids
Source: ISME Commun. 2024 Dec 4;4(1):ycae150. doi: 10.1093/ismeco/ycae150 (PMC11637426; doi:10.1093/ismeco/ycae150)
Supplement: Supplementary_title_text_tables_and_figures_ycae150 [file supplementary_title_text_tables_and_figures_ycae150.pdf]

Supplementary Material for:

Metagenomics reveals the genetic diversity between sublineages of UCYN-A and their algal host plastids

Short Title: **Genome sequences of UCYN-A4 and its algal host plastid**

Ella Joy H. Kantor[1,4], Brent M. Robicheau[2], Jennifer Tolman[1,4], John M. Archibald[3,4], Julie LaRoche[1,4*]

[1]Department of Biology, Dalhousie University, Halifax, NS, Canada

[2]Department of Biology, Clark University, Worcester, MA, USA

[3]Department of Biochemistry & Molecular Biology, Dalhousie University, Halifax, NS, Canada

[4]Institute for Comparative Genomics, Dalhousie University, Halifax, NS, Canada

* Corresponding authors: Julie LaRoche julie.laroche@dal.ca and Ella Joy H. Kantor ella.kantor@dal.ca; Department of Biology, Dalhousie University, Life Sciences Centre, 6287 Alumni Cres, Halifax, NS, B3H 4R2, Canada

The authors declare that they have no competing interests.

Supplementary Information

Supplementary Text:

- Supplementary Methods S1. Commands used to create contigs database
- Supplementary Methods S2. Commands used for Pangenome analysis
- Supplementary Methods S3. Plastid Annotation and Synteny
- Supplementary Methods S3. Plastid Annotation and Synteny
- Supplementary Results S1. Specific details on *nifH* gene region differences
- Supplementary Results S2. 16S rRNA comparisons from *B. bigelowii* plastids

Supplementary Tables:

- Table S1. Information about all genomes in pangenome analysis
- Table S2. Table of UCYN-A enrichment culturing sample methods details.
- Table S3. Metagenomic sequencing details for each enrichment culturing sample.
- Table S4. Table of the top 0.1% of all 16S ASVs used in figures, names, taxonomy, DNA sequence (tableS4.xlsx).
- Table S5. (A) 18S rRNA sequencing results feature table with relative abundance (%) for Basin 2 and Shelf 2 samples and taxonomy classifications from BLAST and Silva. (B) 18S rRNA data used in alignment on sequences from this study and previously published. Accessions, genotype, and sources. (C) Distance matrix of percent identities of alignment (percentage of bases which are identical) (tableS5.xlsx).
- Table S6. CheckM2 Results of genomes in the pangenome analysis
- Table S7. Matrix of ANI values between each genome in the pangenome
- Table S8. Table of unique genes to UCYN-A1 NWA (tableS8_S9.xlsx).
- Table S9. Table of unique genes to UCYN-A4 NWA (tableS8_S9.xlsx).
- Table S10. Distance matrix of UCYN-A16S rRNA gene sequence identities from alignment.
- Table S11. Distance matrix of plastid 16S rRNA gene sequence identities from alignment.

Supplementary Figures:

- Figure S1. Cytograms used during cell sorting of cultures Basin 2 (A, B, C) and Shelf 2 (D, E, F) (note that while Shelf 2 is the sorted cells of the Shelf 1 culture, Basin 2 is not derived from the same water sample as Basin 1). Cell sorting was done by gating for larger particles with higher forward scatter (FSC) values that also encompassed the detection of 3 mm beads within cytograms (Panels A and D). We also required that particles be chlorophyll (chl) positive via higher 692/40 [480] detection (Panels C and F). Phycoerythrin (PE) via florescence at 580/30 [488] (Panels B and E) was also used to try and narrow the sorted population but did not seem to have an impact on the recovery of UCYN-A containing particles – likely due to a very broad gate being used for PE rather than the actual biology of the cells and whether they did or did not have PE. The final nested gates used were "UCYN2" within the broader Chl+/PE+/Large (C+P+L) for Basin 2 and the Chl+/PE-/Large gate (C+P-L) for Shelf 2. The UCYNA2 gate name reflects the name of the qPCR assay that had higher nifH counts when screening populations, but cells were of the UCYN-A4 ecotype.Figure S2. Full tree of all 186 ASVs present in all four enrichment cultures above 0.1% relative abundance, and UCYN-A1 ALOHA and UCYN-A2 CPSB-1 16S sequences for comparison.
- Figure S3. Alignment of NifH amino acid sequences from each of the genomes where *nifH* gene was present.
- Figure S4. Alignment of *B. bigelowii* plastid NWA sequence with published *B. bigelowii* chloroplast sequence from Coale et al. [15] (accessions: OR912955.1, OR912954.1, OR912953.1).

Supplementary References

- [80–96]

Supplementary Methods S1. Commands used to create contigs database:
Prior to the first step in the pipeline, quality filtering of raw reads was done using the 'iu-filter-quality-minoche' program from the illumina-utils library v1.4.1 [80]. Co-assembly was done using MEGAHIT v1.0.3 [81] and mapping of short reads onto contigs was done using Bowtie2 v2.0.5 [82]. Contig fasta files were then reformatted and 'anvi-gen-contigs-database' was further run to generate a contigs database including open reading frames identified by Prodigal v2.6.3 [37,83].

Supplementary Methods S2. Commands used for Pangenome analysis:
Using the contigs database created specifically for the pangenome analysis, a 'genomes storage database' was generated from all the genomes and analysis was run using the command 'anvi-pan-genome' with parameters '--use-ncbi-blast', '--minbit 0.5', and '--mcl-inflation 10'. This program [49] calculates the similarities between genomes, identifies and counts gene clusters, and stores this information in a pan-database to be used for analysis and visualization. Calculation of average nucleotide identity (ANI) was done using the 'anvi-compute-genome-similarity' command with the program PyANI v0.2 [84]. Pangenome visualizations were created using the 'anvi-display-pan' command [49].

Supplementary Methods S3. Plastid Annotation and Synteny:
Both the *B. bigelowii* plastid NWA and *C. parva* genomes were annotated using GeSeq with the tools: ARAGORN v1.2.38, blatN, and blatX [85–87]. To assess synteny various Haptophyte plastid genomes were further aligned using MAUVE v1.1.3 [60] within Geneious using the progressiveMauve algorithm with default settings and then visualized in R (v4.3.0) via RStudio v2023.06.1+524 using the circlize package v0.4.16 [88]. Additionally, Geneious was also used to generate alignment dotplots for the genomes above with parameters set to: include reverse complements, a high sensititve/slow algorithum, score matrix = exact, window size = 100, threshold = 100, and tile size = 2000; the dotplot strategy implements the EMBOSS v6.5.7 dotmatcher [89]. The haptophyte genomes used in these above analyses included: *C. carterae* (NC_069636; [90]), *C. parva* (NC_036937; [52]), *G. huxleyi* (NC_007288; [91]), *G. oceanica* (NC_063782; [92]), and *T. lutea* (NC_040291; [93]]).

Supplementary Methods S3. 16S rRNA data analysis:
16S rRNA amplicon sequencing results were transformed into percent abundances and plotted in R using packages tidyverse v2.0.0 and ggplot2 v3.5.0 [94]. Taxonomic assignment of amplicon sequence variants (ASVs) with >0.1% abundance in each culture was manually refined following the same methods as Robicheau et al. [33]. 16S rRNA amplicon sequence data from the Bedford Basin weekly time series was from the same original dataset and methods as in Robicheau et al. [33] with the exception that all ASVs were retained during analysis rather than subsetting for only plastid signatures. ASVs with >1% relative abundance in the four enrichment cultures were extracted from the time series dataset and plotted to show their annual weekly relative abundance in the natural seawater of the Bedford Basin. A neighbor-joining tree with 1000 bootstraps for 16S rRNA ASVs in the enrichment cultures was built using the online PhyML tool [95] and Muscle v3.8.625 to align sequences [96].

Supplementary Results S1. Specific details on *nifH* gene region differences:
The A2 SIO64986 and A1 ALOHA_A2.5_9 genomes both lack a complete suite of nitrogen fixation genes. The A2 SIO64986 genome does have *nifH*, while in the A1 ALOHA_A2.5_9 genome an obvious *nifH* gene is not present (Figure 4). The *nifH* from the A2 Tara genome is split between two contigs and is missing the portion which aligns to known ASVs or oligotypes. All *nifH* genes found in the UCYN-A1 and -A2 genomes have 100% pairwise identity to the sequences 'A1-deb' and 'Oligo_1' and 'A2-bdd' and 'Oligo_3' [25,12], respectively.
Comparison of the *nifH*-derived amino acid sequences from each genome shows twelve positions with disagreements mainly in the last 10 amino acids (Figure S3). The A1 reference genome's NifH amino acid sequence was identical to that of UCYN-A1 NWA (Figure S3).
Alignment of the full Nif gene region showed a large syntenic block containing 23 genes (Figure 4). There was also one hypothetical protein in the same location for the four published A2 genomes. As mentioned, the

UCYN-A4 NWA has three hypothetical proteins that are not obviously present in any other genomes. For the A2 SIO64986 and A1 ALOHA_A2.5_9 genomes, as in A2 Tara, this region spans multiple contigs, this resulted in missing regions for NifZ in A2 SIO64986, and NifU and NifH in A1 ALOHA_A2.5_9. These fragmented gene regions which are likely due to lower coverage during sequencing and assembly and not an actual missing gene in the genome.

Supplementary Results S2. 16S rRNA comparisons from *B. bigelowi*i plastids:
The 16S rRNA gene from *B. bigelowii* plastid NWA was aligned to other published *B. bigelowii* plastid 16S rRNA genes sequences, as well as to those from other haptophyte plastids and 16S rRNA ASVs obtained herein. All of the B. bigelowii 16S rRNA genes analyzed are >99% identical to each other (Table S11). The *B. bigelowii* plastid NWA 16S rRNA gene was 100% identical to the *B. bigelowii* ASV021, which was in the Shelf 2 and Basin 2 amplicon sequencing results. The ASV020 which was in Shelf 1 and Basin 1 was not 100% identical to any of the others, but it had >99% identity to the other *B. bigelowii* 16S rRNA plastid sequences (Table S11).

Supplementary Tables:

Table S1. Information about all genomes in pangenome analysis

Genome Name	Sublineage	Location	Number of Contigs	Length (bp)	Assembly Accession	File Name	Reference
A1 ALOHA (reference)	UCYN-A1	22.75 N, 158 W	Complete Genome	1443806	GCA_000025125.1		Tripp et al. [18]
A1 ALOHA_A2.5_9	UCYN-A1	22.75 N, 158 W	47	1489669	GCA_022450625.1		Leu et al. [19]
A1 Tara	UCYN- A1	21.4788 S, 56.8291 E	44	1422642		TARA_IOS_50_MAG_00026	Delmont et al. [20]
A2 CPSB-1 (reference)	UCYN- A2	35.5171 N, 133.9374 E	Complete Genome	1491611	GCA_020885515.1		Suzuki et al. [21]
A2 SIO64986	UCYN- A2	32.87 N, 117.25 W	52	1485499	GCA_000737945.1		Bombar et al. [17]
A2 Arc	UCYN- A2	67.1705 N, 0.4423 E	5	1480855		Arc-UCYN-A2	Shiozaki et al. [22]
A2 Tara	UCYN- A2	47.1863 S, 58.2902 W	46	1459650		TARA_AOS_82_MAG_00023	Delmont et al. [20]
UCYN-A1 NWA	UCYN- A1	42.50 N, 61.43 W	7	1437124		RUN1_ALL_MAG_00029	this study
UCYN-A4 NWA	UCYN- A4	42.50 N, 61.43 W	6	1469411		RUN1_ALL_MAG_00024	this study

Table S2. Table of UCYN-A enrichment culturing sample methods details.

Sample		Date collected	Initial Incubation Nutrients	Sorted Cells	Location	Coordinates	Depth	qPCR	Total 16S V6V8
Basin 1	2018_BB_a<br>2018_BB_b<br>2018_BB_c	Aug 22, 2018	2nM Fe + 200nM PO4, 13 weeks at 15℃ with 12h/12h light/dark cycle	No; Seawater incubated with Nutrients straight to sequencing post DNA-extraction	Bedford Basin	44º 41' 37" N, 63º 38' 25" W	1m	Non-specific SYBR qPCR Assay; Had UCYN-A	Similar to A1
Basin 2	2020_BB_a<br>2020_BB_b<br>2020_BB_c	Sept 16, 2020	2nM Fe + 400 nM PO4 + 0.5ml/L vitamins, 2 weeks at 15℃ with 12h/12h light/dark cycle	Cells Sorted (2,000 cells) + Incubation for 8.5 weeks in ~10mL of 0.2um filtered seawater from original sample. Enriched with more nutrients at week 0,2, and 4.	Bedford Basin	44º 41' 37" N, 63º 38' 25" W	5m	Used A1 and A2 TaqMan Assays; Mainly A2	Similar to A2
Shelf 1	2021_Shelf_a	Aug 2021	2nM Fe + 200nM PO4	No; Seawater incubated with Nutrients straight to sequencing post DNA-extraction	Scotian Shelf	42º 50' 00" N, 61º 43' 00" W	20m	Ecotype Specific TaqMan Assays; ~7.5:1 for A1:A2	UCYN-A ASV with higher % similar to A1
Shelf 2	2021_Shelf_b	Aug 2021	2nM Fe + 200nM PO4	Cells sorted without further Incubation (28,911 cells)	Scotian Shelf	42º 50' 00" N, 61º 43' 00" W	20m	Ecotype Specific TaqMan Assays; A2>>A1	Similar to A2

Table S3. Metagenomic sequencing details for each enrichment culturing sample.

Additional sequencing info	Technology - Run	Depth	Date	R1	R2
2018_BB_a	MiSeq - MS251 (S289)	1X	5/3/2019	BBCulture9-20180822_S289_L001_R1_001.fastq.gz	BBCulture9-20180822_S289_L001_R2_001.fastq.gz
2018_BB_b	NextSeq - NS39	4X	5/30/2019	Robichaud_S88_L001_R1_001.fastq.gz, Robichaud_S88_L002_R1_001.fastq.gz, Robichaud_S88_L003_R1_001.fastq.gz, Robichaud_S88_L004_R1_001.fastq.gz	Robichaud_S88_L001_R2_001.fastq.gz, Robichaud_S88_L002_R2_001.fastq.gz, Robichaud_S88_L003_R2_001.fastq.gz, Robichaud_S88_L004_R2_001.fastq.gz
2018_BB_c	MiSeq - MS212 (S385)	1X	12/21/2018	BBculture9_S385_L001_R1_001.fastq.gz	BBculture9_S385_L001_R2_001.fastq.gz
2020_BB_a	NextSeq - NS79 (S42)	4X	2/7/2022	BBc11-20200916-5m-UA2g_S42_L001_R1_001.fastq.gz, BBc11-20200916-5m-UA2g_S42_L002_R1_001.fastq.gz, BBc11-20200916-5m-UA2g_S42_L003_R1_001.fastq.gz, BBc11-20200916-5m-UA2g_S42_L004_R1_001.fastq.gz	BBc11-20200916-5m-UA2g_S42_L001_R2_001.fastq.gz, BBc11-20200916-5m-UA2g_S42_L002_R2_001.fastq.gz, BBc11-20200916-5m-UA2g_S42_L003_R2_001.fastq.gz, BBc11-20200916-5m-UA2g_S42_L004_R2_001.fastq.gz
2020_BB_b	NextSeq - NS83	4X	3/16/2022	BBc11-20200916-5m-UA2g_S46_L001_R1_001.fastq.gz, BBc11-20200916-5m-UA2g_S46_L002_R1_001.fastq.gz, BBc11-20200916-5m-UA2g_S46_L003_R1_001.fastq.gz, BBc11-20200916-5m-UA2g_S46_L004_R1_001.fastq.gz	BBc11-20200916-5m-UA2g_S46_L001_R2_001.fastq.gz, BBc11-20200916-5m-UA2g_S46_L002_R2_001.fastq.gz, BBc11-20200916-5m-UA2g_S46_L003_R2_001.fastq.gz, BBc11-20200916-5m-UA2g_S46_L004_R2_001.fastq.gz
2020_BB_c	NextSeq - NS72 (S69)	1X	8/27/2021	BBc11_20200916_5m_UA2g_S69_L001_R1_001.fastq.gz, BBc11_20200916_5m_UA2g_S69_L002_R1_001.fastq.gz, BBc11_20200916_5m_UA2g_S69_L003_R1_001.fastq.gz, BBc11_20200916_5m_UA2g_S69_L004_R1_001.fastq.gz	BBc11_20200916_5m_UA2g_S69_L001_R2_001.fastq.gz, BBc11_20200916_5m_UA2g_S69_L002_R2_001.fastq.gz, BBc11_20200916_5m_UA2g_S69_L003_R2_001.fastq.gz, BBc11_20200916_5m_UA2g_S69_L004_R2_001.fastq.gz
2021_Shelf_a	NextSeq - NS99	6X	12/6/2022	BR2021_HL7_20m_enriched_S86_L001_R1_001.fastq.gz	BR2021_HL7_20m_enriched_S86_L001_R2_001.fastq.gz
2021_Shelf_b	NextSeq - NS99	6X	12/6/2022	JA_SortedCells_UA2_rep10_S87_L001_R1_001.fastq.gz	JA_SortedCells_UA2_rep10_S87_L001_R2_001.fastq.gz

Table S4. Table of the top 0.1% of all 16S ASVs used in figures, names, taxonomy, DNA sequence (tableS4.xlsx).

Table S5. (A) 18S rRNA sequencing results feature table with relative abundance (percent) for Basin 2 and Shelf 2 samples and taxonomy classifications from BLAST and Silva. (B) 18S rRNA data used in alignment. Accessions, genotype, sources. (C) Distance matrix of identities of alignment (tableS5.xlsx).

Table S6. CheckM2 results of genomes in the pangenome analysis

Genome Name	Completeness	Contamination	Completeness Model Used	Translation Table Used	Coding Density	Contig N50	Average Gene Length	Genome Size	GC Content	Total Coding Sequences	Additional Notes
A1 ALOHA_2.5_9	99.22	0.52	Neural Network (Specific Model)	11	0.817	86550	316.285047	1489669	0.31	1284	None
A1 Tara	98.2	0.19	Neural Network (Specific Model)	11	0.816	51922	320.384934	1422642	0.31	1208	None
A1 reference (ALOHA)	99.58	0.09	Neural Network (Specific Model)	11	0.808	1443806	325.370401	1443806	0.31	1196	None
A2 Arc	99.17	0.1	Neural Network (Specific Model)	11	0.791	364024	321.712521	1480855	0.31	1214	None
A2 SIO64986	98.62	0.09	Neural Network (Specific Model)	11	0.785	73988	311.590545	1485499	0.31	1248	None
A2 reference (CPSB-1)	99.45	0.09	Neural Network (Specific Model)	11	0.785	1491611	322.82562	1491611	0.31	1210	None
A2 Tara	96.47	0.38	Neural Network (Specific Model)	11	0.788	44130	316.413366	1459650	0.31	1212	None
UCYN-A4 NWA	99.35	0.08	Neural Network (Specific Model)	11	0.802	361971	325.426325	1469411	0.31	1208	None
UCYN-A1 NWA	99.16	0.09	Neural Network (Specific Model)	11	0.817	352113	327.367057	1437124	0.31	1196	None

Table S7. Matrix of ANI values between each genome in the pangenome analysis.

Genome Name	A1 ALOHA_A2.5_9	A1 Tara	A1 reference (ALOHA)	A2 reference (CPSB-1)	A2 SIO64986	A2 Arc	A2 Tara	UCYN-A1 NWA	UCYN-A4 NWA
**A1 ALOHA_A2.5_9**	100	99.25	99.93	83.26	83.34	83.37	83.24	99.90	82.76
**A1 Tara**	99.23	100	99.26	83.30	83.30	83.34	83.29	99.28	82.74
**A1 reference (ALOHA)**	99.92	99.28	100	83.32	83.44	83.46	83.28	99.91	82.72
**A2 Arc**	83.27	83.32	83.33	100	99.63	99.33	99.74	83.33	85.34
**A2 SIO64986**	83.37	83.31	83.42	99.62	100	99.26	99.65	83.31	85.33
**A2 reference (CPSB-1)**	83.38	83.34	83.41	99.33	99.25	100	99.31	83.32	85.40
**A2 Tara**	83.31	83.35	83.36	99.73	99.64	99.30	100	83.36	85.35
**UCYN-A1 NWA**	99.90	99.30	99.91	83.27	83.26	83.28	83.28	100	82.75
**UCYN-A4 NWA**	82.60	82.69	82.65	85.26	85.31	85.28	85.33	82.65	100

Table S8. Unique genes to the A1 MAG (tableS8_S9_uniqueGenes.xlsx).

Table S9. Unique genes to the A4 MAG (tableS8_S9_uniqueGenes.xlsx).

Table S10. Distance matrix of UCYN-A16S rRNA SSU gene sequence percent identities from alignment.

**16S Sequence**	**A2 CPSB-1 (reference) 16S (forward)**	**A1 ALOHA_A 2.5_9 16S**	**A1 ALOHA (reference) 16S**	**Basin 1 16S V6-V8 ASV022***	**A3 (MH807559)-partial 16S**	**A2 TMRscBb7 - 16S (AB847982)**	**A2 SIO64986 - 16S**	**A2 reference (CPSB-1) 16S (reverse)**	**Shelf 2 16S V6-V8 ASV023***
**A2 CPSB-1 (reference) 16S (forward)**		88.072	88.067	80.789	86.825	87.606	88.27	88.337	81.316
**A1 ALOHA_A2.5_9 16S**	88.072		100	100	98.946	98.528	98.662	98.654	97.368
**A1 ALOHA (reference) 16S**	88.067	100		100	98.946	98.528	98.714	98.714	97.368
**Basin 1 16S V6-V8 ASV022***	80.789	100	100		97.632	98.158	98.158	98.158	97.368
**A3 (MH807559)-partial 16S**	86.825	98.946	98.946	97.632		98.588	98.645	98.72	99.211
**A2 TMRscBb7-16S (AB847982)**	87.606	98.528	98.528	98.158	98.588		99.892	99.821	99.211
**A2 SIO64986 - 16S**	88.27	98.662	98.714	98.158	98.645	99.892		99.933	99.211
**A2 reference (CPSB-1) 16S (reverse)**	88.337	98.654	98.714	98.158	98.72	99.821	99.933		99.211
**Shelf 2 16S V6-V8 ASV023***	81.316	97.368	97.368	97.368	99.211	99.211	99.211	99.211	

Table S11. Distance matrix of plastid 16S rRNA gene sequence identities from alignment (*this study).

**Plastid 16S rRNA sequence**	**KJ201907 – C. tobinii 16S rRNA**	**NC_036937 – C. parva 16S rRNA**	**AY741371 – G. hux 16S rRNA**	**LC595682 – NIES-4442**	**B. bigelowii NWA - 16S rRNA***	**16S ASV021 B. bigelowii***	**AB847984 – isolate TMRscBb1**	**AB847985 – isolate TMRscBb7**	**AB847986 – isolate TMRscBb8**	**16S ASV020 – B. bigelowii***
**KJ201907 – C. tobinii 16S rRNA**		100	94.494	94.868	94.969	91.579	94.873	94.895	94.873	91.316
**NC_036937 – C. parva 16S rRNA**	100		94.494	94.868	94.963	91.579	94.873	94.895	94.873	91.316
**AY741371 – G. hux 16S rRNA**	94.494	94.494		95.423	95.649	92.368	95.745	95.694	95.673	92.632
**LC595682 – NIES-4442**	94.868	94.868	95.423		99.584	99.474	99.963	99.963	99.963	99.737
**B. bigelowii NWA- 16S rRNA***	94.969	94.963	95.649	99.584		100	99.673	99.691	99.673	99.737
**16S ASV021 – B. bigelowii***	91.579	91.579	92.368	99.474	100		99.474	99.474	99.474	99.737
**AB847984 – isolate TMRscBb1**	94.873	94.873	95.745	99.963	99.673	99.474		99.909	99.964	99.737
**AB847985 – isolate TMRscBb7**	94.895	94.895	95.694	99.963	99.691	99.474	99.909		99.909	99.737
**AB847986 – isolate TMRscBb8**	94.873	94.873	95.673	99.963	99.673	99.474	99.964	99.909		99.737
**16S ASV020 – B. bigelowii***	91.316	91.316	92.632	99.737	99.737	99.737	99.737	99.737	99.737	

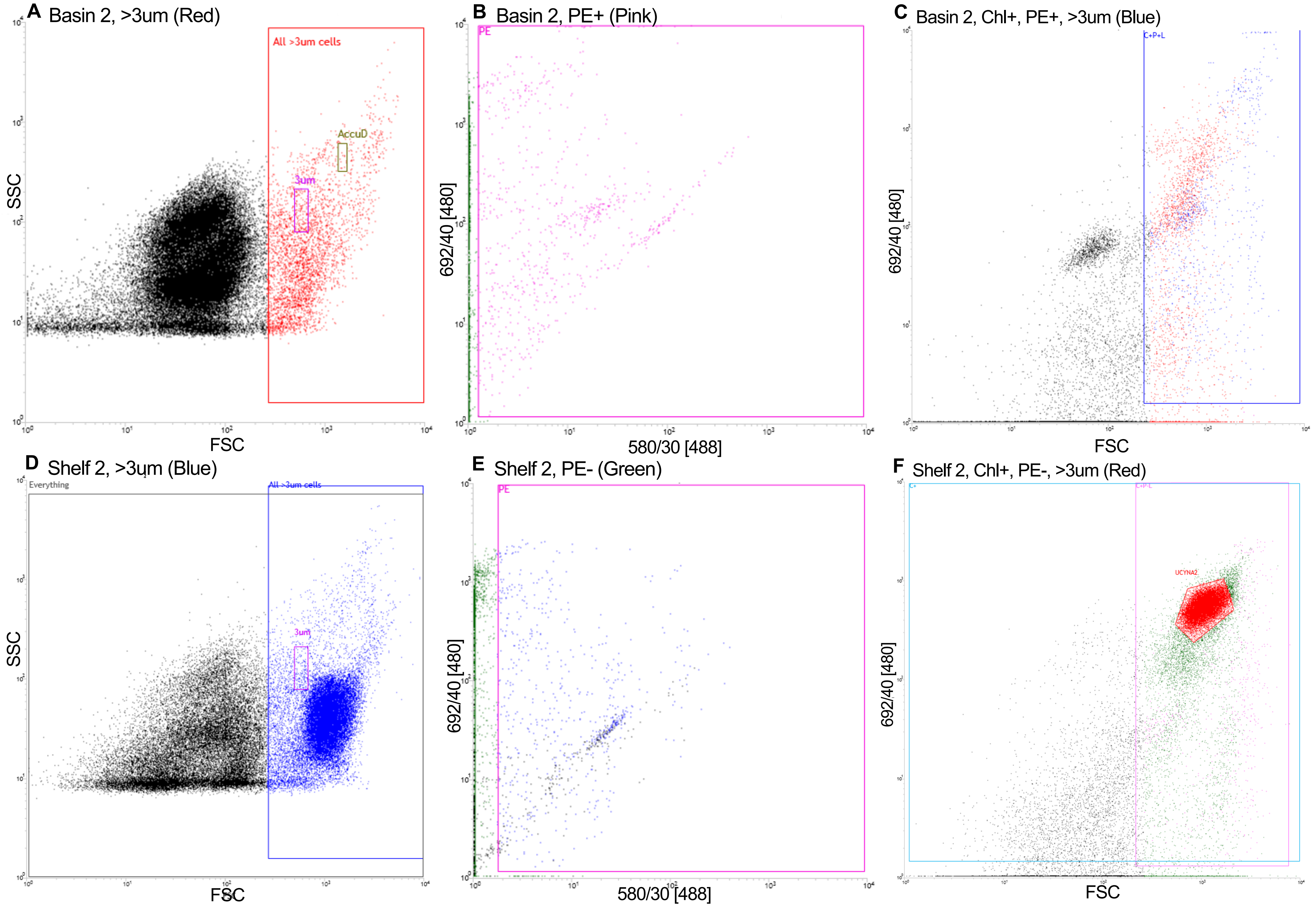

Figure S1. Cytograms used during cell sorting of cultures Basin 2 (A, B, C) and Shelf 2 (D, E, F) (note that while Shelf 2 is the sorted cells of the Shelf 1 culture, Basin 2 is not derived from the same water sample as Basin 1). Cell sorting was done by gating for larger particles with higher forward scatter (FSC) values that also encompassed the detection of 3 mm beads within cytograms (Panels A and D). We also required that particles be chlorophyll (chl) positive via higher 692/40 [480] detection (Panels C and F). Phycoerythrin (PE) via florescence at 580/30 [488] (Panels B and E) was also used to try and narrow the sorted population but did not seem to have an impact on the recovery of UCYN-A containing particles – likely due to a very broad gate being used for PE rather than the actual biology of the cells and whether they did or did not have PE. The final nested gates used were "UCYN2" within the broader Chl+/PE+/Large (C+P+L) for Basin 2 and the Chl+/PE-/Large gate (C+P-L) for Shelf 2. The UCYNA2 gate name reflects the name of the qPCR assay that had higher nifH counts when screening populations, but cells were of the UCYN-A4 ecotype.

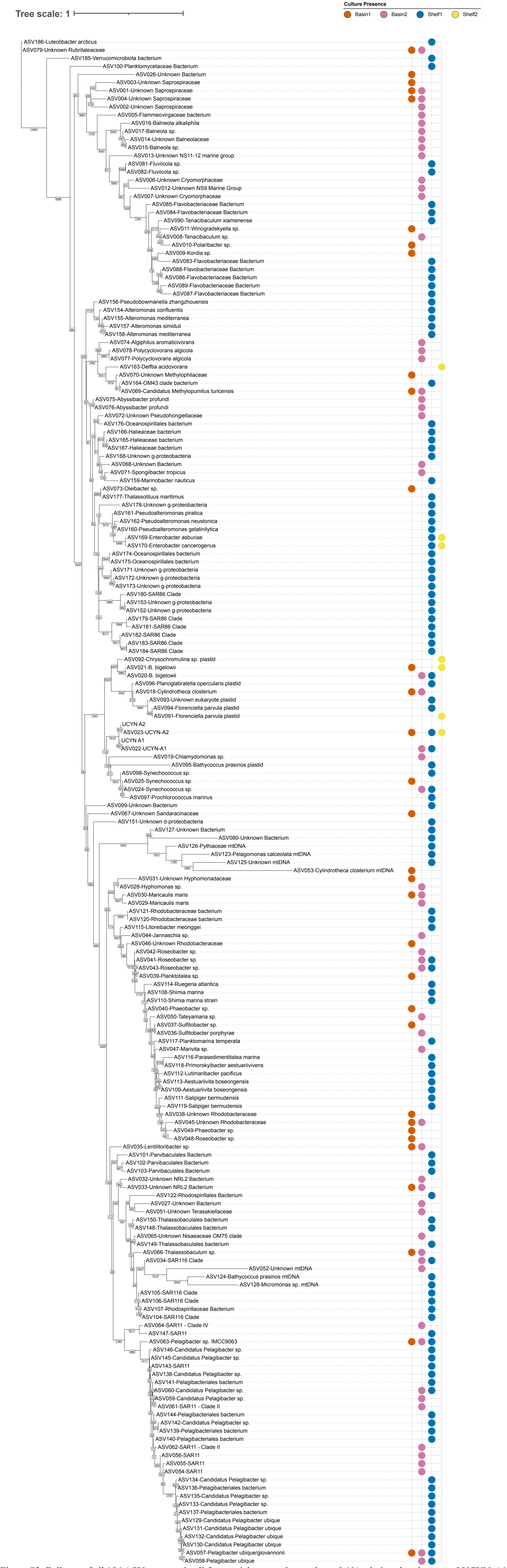

Figure S2. Full tree of all 186 ASVs present in all four enrichment cultures above 0.1% relative abundance, and UCYN-A1 ALOHA and UCYN-A2 CPSB-1 16S sequences for comparison.

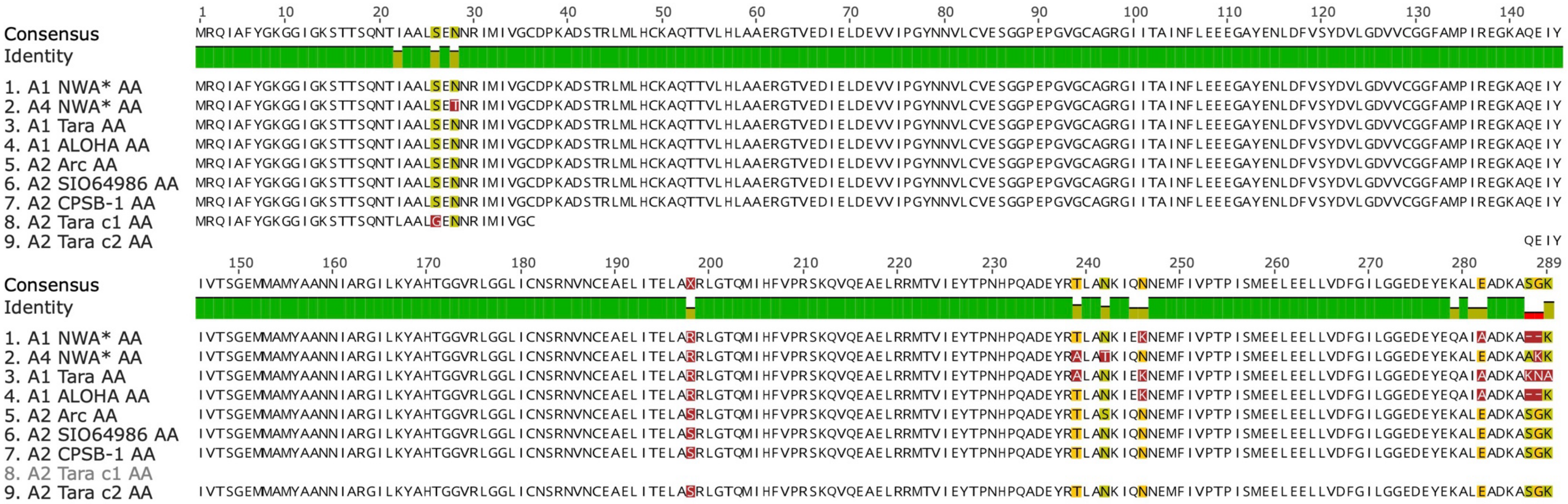

Figure S3. Alignment of NifH amino acid sequences from each of the genomes where *nifH* gene was present.

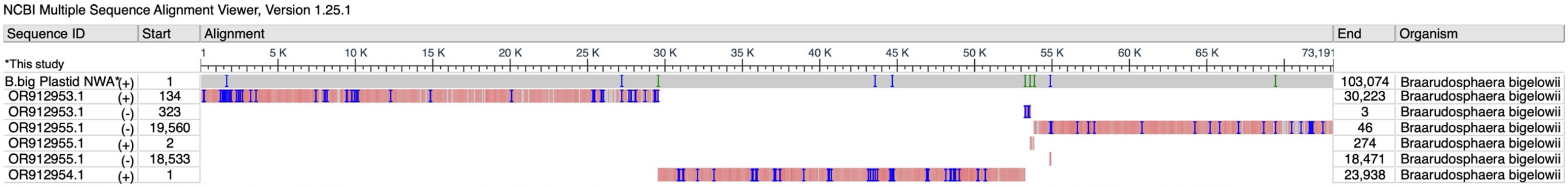

Figure S4. Alignment of *B. bigelowii* plastid NWA sequence with published *B. bigelowii* chloroplast sequence from Coale et al. [15] (acc: OR912955.1, OR912954.1, OR912953.1).

Supplementary References

80. Eren AM, Vineis JH, Morrison HG, Sogin ML. A Filtering Method to Generate High Quality Short Reads Using Illumina Paired-End Technology. *PLOS ONE* 2013;**8**:e66643.

81. Li D, Liu C-M, Luo R, Sadakane K, Lam T-W. MEGAHIT: an ultra-fast single-node solution for large and complex metagenomics assembly via succinct de Bruijn graph. *Bioinformatics* 2015;**31**:1674–6.

82. Langmead B, Wilks C, Antonescu V, Charles R. Scaling read aligners to hundreds of threads on general-purpose processors. *Bioinformatics* 2019;**35**:421–32.

83. Hyatt D, Chen G-L, LoCascio PF, Land ML, Larimer FW, Hauser LJ. Prodigal: prokaryotic gene recognition and translation initiation site identification. *BMC Bioinformatics* 2010;**11**:119.

84. Pritchard L, Glover RH, Humphris S, Elphinstone JG, Toth IK. Genomics and taxonomy in diagnostics for food security: soft-rotting enterobacterial plant pathogens. *Anal Methods* 2015;**8**:12–24.

85. Tillich M, Lehwark P, Pellizzer T, Ulbricht-Jones ES, Fischer A, Bock R *et al.* GeSeq – versatile and accurate annotation of organelle genomes. *Nucleic Acids Research* 2017;**45**:W6–11.

86. Laslett D, Canback B. ARAGORN, a program to detect tRNA genes and tmRNA genes in nucleotide sequences. *Nucleic Acids Research* 2004;**32**:11–6.

87. Kent WJ. BLAT—The BLAST-Like Alignment Tool. *Genome Res* 2002;**12**:656–64.

88. Gu Z, Gu L, Eils R, Schlesner M, Brors B. circlize Implements and enhances circular visualization in R. *Bioinformatics* 2014;**30**:2811–2.

89. Rice P, Longden I, Bleasby A. EMBOSS: the European Molecular Biology Open Software Suite. *Trends Genet* 2000;**16**:276–7.

90. Chrysotila carterae chloroplast, complete genome. 2023.

91. Sánchez Puerta MV, Bachvaroff TR, Delwiche CF. The complete plastid genome sequence of the haptophyte Emiliania huxleyi: a comparison to other plastid genomes. *DNA Res* 2005;**12**:151–6.

92. Kao T-T, Wang T-H, Ku C. Rampant nuclear–mitochondrial–plastid phylogenomic discordance in globally distributed calcifying microalgae. *New Phytologist* 2022;**235**:1394–408.

93. Méndez-Leyva AB, Guo J, Mudd EA, Wong J, Schwartz J-M, Day A. The chloroplast genome of the marine microalga Tisochrysis lutea. *Mitochondrial DNA Part B* 2019;**4**:253–5.

94. Wickham H, Averick M, Bryan J, Chang W, McGowen LD'A, François R *et al.* Welcome to the Tidyverse. *Journal of Open Source Software* 2019;**4**:1686.

95. Guindon S, Dufayard J-F, Lefort V, Anisimova M, Hordijk W, Gascuel O. New Algorithms and Methods to Estimate Maximum-Likelihood Phylogenies: Assessing the Performance of PhyML 3.0. *Systematic Biology* 2010;**59**:307–21.

96. Edgar RC. MUSCLE: a multiple sequence alignment method with reduced time and space complexity. *BMC Bioinformatics* 2004;**5**:113.
